# High-precision intermode beating electro-optic distance measurement for mitigation of atmospheric delays

**DOI:** 10.1515/jag-2022-0039

**Published:** 2023-04-25

**Authors:** Pabitro Ray, David Salido-Monzú, Andreas Wieser

**Affiliations:** Institute of Geodesy and Photogrammetry, ETH Zurich, Stefano-Franscini-Platz 5, 8093 Zurich, Switzerland

**Keywords:** frequency combs, multiwavelength EDM, optical metrology, refractivity compensation, supercontinuum

## Abstract

High-precision electro-optic distance measurement (EDM) is essential for deformation monitoring. Although sub-ppm instrumental accuracy is already feasible with state-of-the-art commercial technology, the practically attainable accuracy on distances over more than a few hundred meters is limited by uncertainties in estimating the integral refractive index along the propagation path, which often results in measurement errors of several ppm. This paper presents a new instrumental basis for high-accuracy multispectral EDM using an optical supercontinuum to enable dispersion-based inline refractivity compensation. Initial experiments performed on two spectrally filtered bands of 590 and 890 nm from the supercontinuum show measurement precision better than 0.05 mm over 50 m for an acquisition time of around 3 ms on the individual bands. This represents a comparable performance to our previously reported results on 5 cm by over a range of 3 orders of magnitude longer, which can still be improved by increasing the acquisition time. The preliminary results indicate a relative accuracy of about 0.1 mm at 50 m on each wavelength. Improvement is possible by calibration and by implementing a self-reference scheme that mitigates slow drifts caused by power-to-phase coupling. The results reported herein thus indicate that the presented approach can be further developed for achieving sub-ppm accuracy of refractivity compensated distance measurements on practically useful ranges and under outdoor conditions.

## 1 Introduction

High-precision electro-optic distance measurement (EDM) technology is a key element for a variety of applications such as surveying, industrial metrology, and deformation monitoring of large-scale structures [1]. The technology has enabled non-contact measurements over distances from a few mm to several km with sub-mm-level instrumental precision. However, for long-distance EDM (distances > 100 m), the measurement accuracy under practical conditions is limited by the uncertainties of the refractive index (*n*) of the air. Errors result from insufficient knowledge of the atmospheric conditions and thus of *n* along the optical path. This restricts the accuracy of long-distance EDM to 1 ppm or worse with commercial instruments [2]. For standard air and EDM using optical wavelengths, primarily spatial and temporal variations of temperature (*T*) and pressure (*P*) cause refractivity errors. E.g., a deviation of 1 K in temperature or 4 hPa in pressure leads to a distance deviation of 1 ppm. Relative humidity (RH) and CO_2_ concentration (*X*_c_) have a much smaller impact.

Conventionally, such refractivity-induced errors are mitigated by forward modeling using meteorological parameters measured at a few locations (often only at the instrument location) and estimating the refractive index using empirical equations such as Ciddor’s equations [3, 4] or Edlen’s updated equations [5]. The accuracy of these empirical equations is on the order of 10^−8^, representing the best achievable performance for distance measurements in standard air [6]. While this method works well for relatively short distances, discrete sets of meteorological observations typically fail to represent the true spatial distribution of refractivity along the optical path over longer distances. Recent research has shown that even taking a dense set of meteorological measurements along the entire path (e.g., simultaneously every 10 m along a 600 m line-of-sight), achieving distance corrections below the ppm level is exceptionally challenging under practical outdoor conditions with forward modeling [7]. Therefore, achieving high accuracy EDM over long distances requires employing a method capable of self-correcting for refractivity errors, thereby alleviating the necessity to estimate the refractivity from meteorological parameters.

Dual-wavelength compensation (traditionally referred to as the two-color method) is a well-known technique that allows self-correction of refractivity variations using simultaneously acquired distance measurements obtained using optical radiation at two different wavelengths [8]. The technique achieves inline refractivity compensation through the differential delay caused by dispersion. The first experimental demonstration of dual-wavelength compensation was presented over 50 years ago using a He–Ne laser at 632.8 nm and light at 368.1 nm filtered from a mercury arc lamp [9]. Based on the same principle, the commercial instrument Terrameter was later conceived using a He–Ne (633 nm) and a He–Cd (440 nm) laser [10]. However, limitations imposed by the technical challenges of the setup made this commercial realization impractical, and the necessary fractional stability (Δ*f*/*f*) of the laser frequency (*f*) prevented the achievable accuracy from getting close to the accuracy level of the empirical equations.

In recent years, with the discovery of frequency combs [11], the fractional stability of the laser frequency has improved to the precision levels of a Rubidium (Rb) atomic clock, i.e., Δ*f*/*f* ≈ 10^−11^ or even better [12]. This significant improvement, along with concurrent advances in spectral manipulation technologies and high-frequency/low-noise electronic instrumentation [13], has provided a foundation for pushing EDM accuracy further.

Following the pioneering work by [14] on EDM using a frequency comb derived from a mode-locked femtosecond (fs) laser, numerous investigations have continually improved the state-of-the-art of precision distance metrology [15–17]. Inline refractivity-compensated EDM using dual-wavelength frequency combs has been reported [6, 12, 18, 19]. However, in general, two fixed-wavelength combs are used (usually around the standard telecommunication wavelength of around 1560 nm and its frequency multiplied components, e.g., 780 nm), which restricts the possible spectral combinations and requires co-aligning of different beams.

In this work, we use a 420 nm wide supercontinuum (SC) coherently broadened from a 780 nm mode-locked fs laser. The broad spectral range of the SC, ranging from 580 to 1000 nm, allows more flexibility in selecting the optimal combination of wavelengths, possibly even more than two from the entire span of the spectrum. Additionally, compared to the traditional comb sources, the SC offers increased flexibility in gathering power and phase signatures over a wide range of available wavelengths, representing a suitable source to enable dispersion-based inline refractivity compensation through multiwavelength EDM. The experimental feasibility of this approach has been previously demonstrated over very short distances of only about 5 cm [20]. In this paper, we extend the previous work by demonstrating SC-based dual-wavelength EDM over 50 m, i.e., increasing the distance by 3 orders of magnitude. The experimental platform is already designed such as to be later adaptable for distances again longer by 1–2 orders of magnitude and thus for surveying and monitoring applications.

The remainder of the paper is organized as follows: we give a brief introduction to the dual-wavelength method and describe intermode beating. We then explain the experimental setup and results for dual-wavelength measurements over 50 m. Finally, we summarize and conclude with key areas to be investigated for improving the present performance.

## 2 Approach

### 2.1 Dual-wavelength method

**Figure 1:**
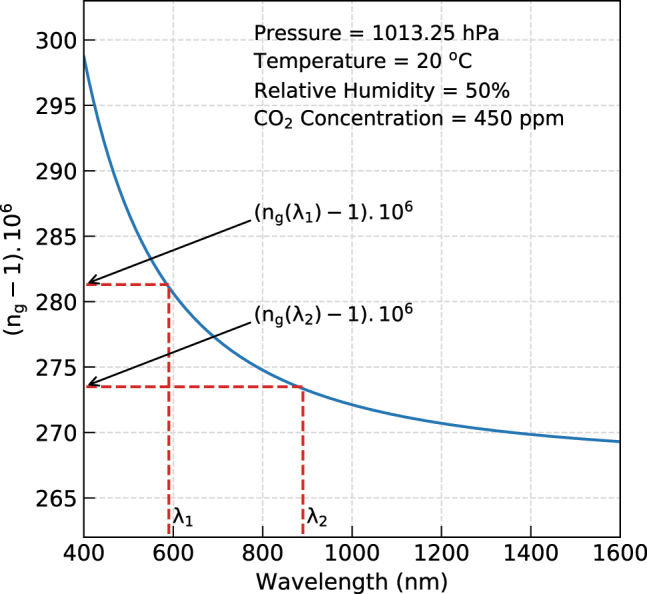
Dispersion profile of standard air over 400–1600 nm. Group refractivity indices for the wavelength pair 590/890 nm.

The atmosphere is dispersive for optical waves, i.e., the refractivity depends on the wavelength. Figure 1 shows this for the group refractivity (*n*_g_ − 1) and wavelengths between 400 and 1600 nm.

By exploiting dispersion, the true geometrical distance (*D*) can be determined from simultaneous distance measurements, i.e., measurements of the optical path length (*L*_1_, *L*_2_) on two wavelengths (*λ*_1_, *λ*_2_) as

(1) $$D=L_{1}-A\left(L_{2}-L_{1}\right)$$

where *A* = (*n*_1_ − 1)/(*n*_2_ − *n*_1_) is the dispersion-coefficient; *n*_*i*_ is the average refractive index of air along the measurement path at the wavelength *λ*_*i*_ and the optical path length is related to the true length by *L*_*i*_ = *n*_*i*_*D*. In the case of dry air, *A* is very weakly dependent on the environmental parameters and can be assumed constant under practical conditions. E.g., for the set of parameters in Figure 1 with RH = 0% and any wavelength pair in the range from 400 to 1600 nm, changes of Δ*T* = 10 K or Δ*P* = 40 hPa introduce relative changes of around 2 ppm in *A*, causing negligible relative errors of the estimated distance (below 10^−9^). For wet air with RH = 50%, the dependence of *A* on *T* and *P* increases, and the same changes in Δ*T* = 10 K or Δ*P* = 40 hPa introduce relative distance errors of around 1 ppm and 0.05 ppm respectively. The impact of Δ*X*_c_ on relative distance errors can be neglected. However, the dependence of *A* on RH is also relevant. E.g., for the set of parameters in Figure 1 and any wavelength pair in the above range, a change ΔRH = 40% results in relative distance errors of close to 1 ppm. This shows a practical limitation of the method, but it also indicates that inline atmospheric compensation with an accuracy of about 1 ppm or better is possible with coarse meteorological measurements at the instrument location or even just assumptions on *P* and *X*_c_. Sub-ppm level accuracy using the two-color method requires a good approximation of *T* and RH along the propagation path for appropriately computing the *A* factor and the corrected distance.

An important design choice is the selection of the two wavelengths which determines the value of *A*. Some approximate values of *A* for different wavelength pairs are listed in Table 1 (the values in the table and thereafter are calculated using Ciddor’s group refractive index according to the corrected equations and implementation provided in the literature [21]). Taking these values and (1) into account, it can be deduced that any measurement noise of the difference between *L*_1_ and *L*_2_ is scaled up significantly, resulting in a higher standard deviation of the atmospherically corrected distance (*D*) than of the individual measurements *L*_1_ and *L*_2_. Thus, it is desirable to select the wavelength pair such that the value of *A* is as low as possible given the technological constraints of the instrument (e.g., the possible range of wavelengths, different atmospheric attenuation at different wavelengths, and different measurement noise). This implies that the two wavelengths should be far separated from each other around the steeper gradient region of the dispersion curve while avoiding spectral regions (e.g., too close to the ultraviolet regime) with excessive atmospheric absorption or scattering.

**Table 1:** Dispersion coefficient for different wavelengths.

Wavelength pairs (nm/nm)	|*A*| calculated from *n*_g_
1560/780	47
1560/590	23
890/590	35

### 2.2 Intermode beating

**Figure 2:**
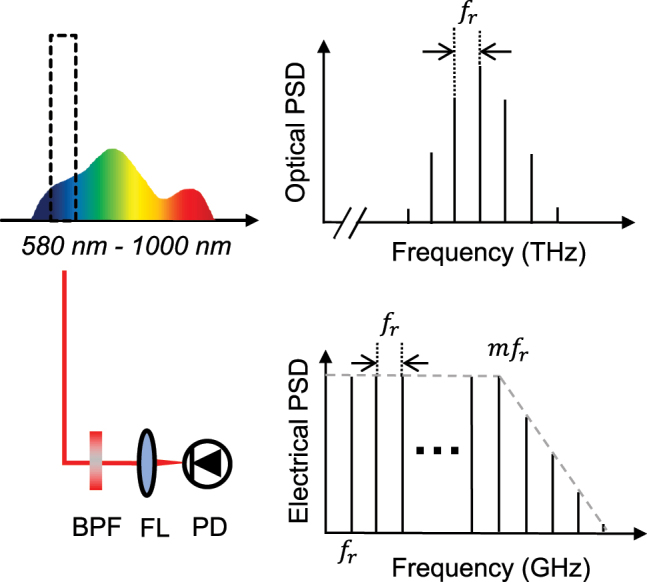
Schematic representation of the optical comb from the filtered spectrum of the supercontinuum and the intermode beat notes constituting the electrical comb (BPF, band-pass filter; FL, focusing lens; PD, photodetector; PSD, power spectral density).

The measurement principle that we use for the experimental setup relies on intermode beating [14, 17], where the beat notes are generated by direct photodetection of a filtered region of an SC. This technique assures a traceable link between the optical (THz) and radio (GHz) frequencies with high accuracy [11]. For example, if the frequency spacing of each comb line in the optical domain is *f*_r_, the adjacent comb lines from filtered SC interfere with each other at the photodetector (PD), resulting in an electrical comb spectrum of *mf*_r_ where *m* = 1, 2, 3, … and limited by the PD bandwidth. Figure 2 shows the optical power spectral density (PSD) of the filtered SC and the generated electrical comb PSD from direct photodetection.

A key advantage of this approach is that the individual intermode beat notes can be used for EDM like different measurement wavelengths in conventional phase-based EDM but without the need for actual modulators [17]. Besides, the frequency stability of beat notes is similar to that of the original comb and is therefore suitable for delivering high-accuracy EDM.

Our measurements are derived from the phase difference (Δ*ϕ* = *ϕ* − *ϕ*_ref_) of one of the beat notes *m*′*f*_r_ over a measurement path of length *L* with respect to a fixed reference path *L*_ref_. Omitting phase ambiguity for simplicity, the measured phases are

(2) $$\phi \left(m^{\prime }f_{\text{r}}\right)=\left(2\pi n_{\text{g}}^{\lambda }/c\right)m^{\prime }f_{\text{r}}L+\phi_{0}$$

(3) $$\phi_{\text{ref}}\left(m^{\prime }f_{\text{r}}\right)=\left(2\pi n_{\text{g}}^{\lambda }/c\right)m^{\prime }f_{\text{r}}L_{\text{ref}}+\phi_{0}$$

Where, $n_{\text{g}}^{\lambda }$ is the group refractive index of air at *λ*, *c* is the speed of light in vacuum, and *ϕ*_0_ is the common phase offset for the two paths. The differential path length (Δ*L* = *L* − *L*_ref_) is then estimated from the phase difference $\Delta \phi \left(m^{\prime }f_{\text{r}}\right)=\phi \left(m^{\prime }f_{\text{r}}\right)-\phi_{\text{ref}}\left(m^{\prime }f_{\text{r}}\right)$ as

(4) $$\Delta L=\left(c/2\pi n_{\text{g}}^{\lambda }m^{\prime }f_{\text{r}}\right)\Delta \phi \left(m^{\prime }f_{\text{r}}\right)$$

In general, higher-order beat notes are helpful to obtain higher distance precision for the same signal-to-noise ratio (SNR) due to the smaller scaling between phase and distance. The maximum beat note frequency is only defined by the PD bandwidth. Higher beat notes can therefore be accessed by selecting a faster PD as far as the current photodetector technology allows.

Besides the intermode beating approach, several other interferometric [12, 22, 23], advanced time-of-flight [24], and hybrid methods combining the interferometric phase and delay-based approaches [16] have also been reported in the literature. Although the sensitivity and precision of intermode beating-based EDM are not as high as the interferometric methods, this approach is robust against practical environmental fluctuations [20] hence adequate for long-distance measurements. Recent research has also shown that it is a promising alternative to other established EDM techniques such as time-of-flight or amplitude modulated phase-based EDM [14, 17].

## 3 Experimental setup

**Figure 3:**
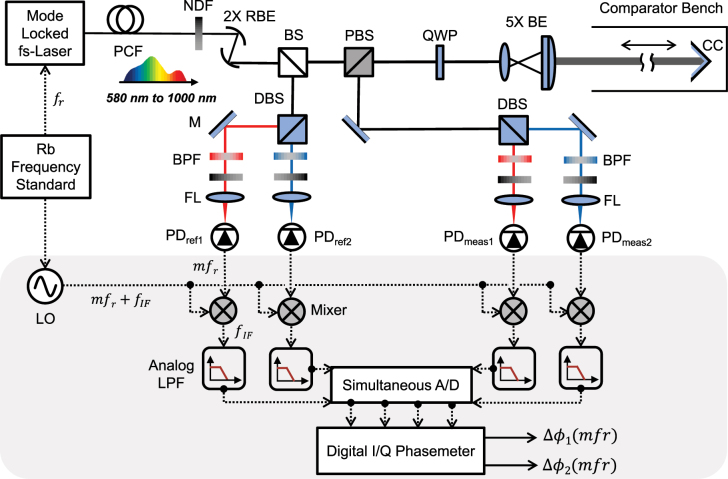
Diagram of the experimental setup (PCF, photonic crystal fiber; NDF, neutral density filter; RBE, reflective beam expander; BS, beam splitter; PBS, polarization beam splitter; QWP, quarter-wave plate; BE, beam expander; CC, corner cube; DBS, dichroic beam splitter; M, plane mirror; BPF, band-pass filter; FL, focusing lens; PD, photodetector; LO, local oscillator; LPF, low-pass filter; A/D, analog-to-digital; I/Q, inline-quadrature).

We have developed and calibrated an experimental platform implementing intermode beating based simultaneous EDM on two spectral bands derived from an fs-laser coherent supercontinuum (SC). A schematic of this setup is shown in Figure 3. We use a 420 nm wide supercontinuum (SC) source spanning wavelengths from 580 nm to 1000 nm having a total optical power of slightly over 50 mW. The SC is derived from a frequency stabilized 780 nm mode-locked fs-laser (Menlo Systems C-fiber 780 SYNC100), spectrally broadened through a photonic crystal fiber (PCF) (Menlo Systems SCG1500). A Rubidium (Rb) frequency standard (SRS FS725) is used for internal stabilization of the 100 MHz pulse repetition frequency *f*_r_ of the laser and as the time basis for the mixing and acquisition electronics.

**Figure 4:**
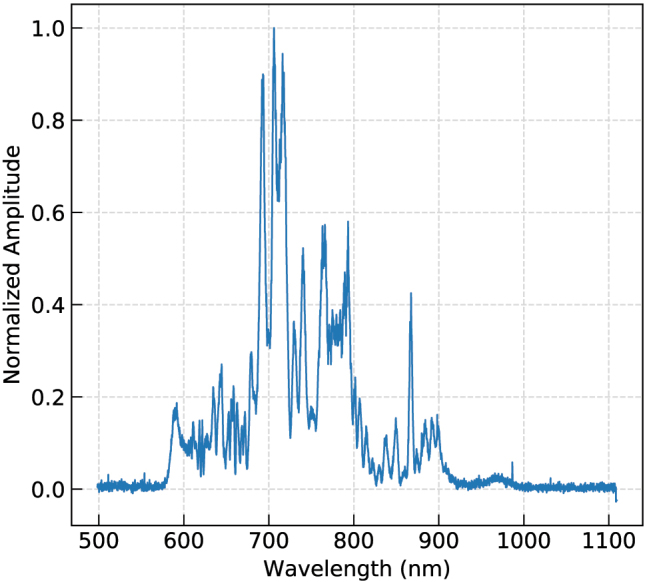
Instantaneous spectrum of the supercontinuum.

Figure 4 shows the optical spectrum of the generated SC as indicated by a Thorlabs CCS175 spectrometer. Although the average power of the SC is stable, its power spectral density is affected by significant temporal variations due to the high sensitivity of the seed 780 nm laser to thermal effects and optical changes (e.g., power, pointing, polarization) [25].

The wavelength pair and spectral bandwidth selection for the dual-wavelength measurements should therefore not only consider the dispersion factor (*A*) favoring wavelengths separated as far as possible, but also the spectral stability over time, and the integrated power per band defining the SNR of the generated beat notes. Taking these aspects into account, along with observations of our actual SC spectrum and its variability over time, we have selected two stable regions around 590 nm and 890 nm, extracted from the SC by band-pass filtering with 10 nm width right before photodetection (see Figure 3).

These filtered bands from the reference and measurement paths are fed to individual high-speed avalanche photodiodes (Menlo Systems APD 210). The intermode beat notes generated at the detector are separated by 100 MHz (corresponding to the pulse repetition frequency of the laser), with a relatively constant power within the photodiode bandwidth up to 1 GHz. Our experiments use the phase-delay information on the 1 GHz beat note, which is the fastest available mode still providing high SNR. The 1 GHz intermode beat is then down-converted to an intermediate frequency *f*_IF_ = 1 MHz and acquired on a simultaneous digitizer at a sampling rate of 1.25 GS/s after anti-aliasing filtering. The phase difference between the reference and measurement paths per spectral band is estimated via a digital Inline-Quadrature (I/Q) phasemeter.

**Figure 5:**
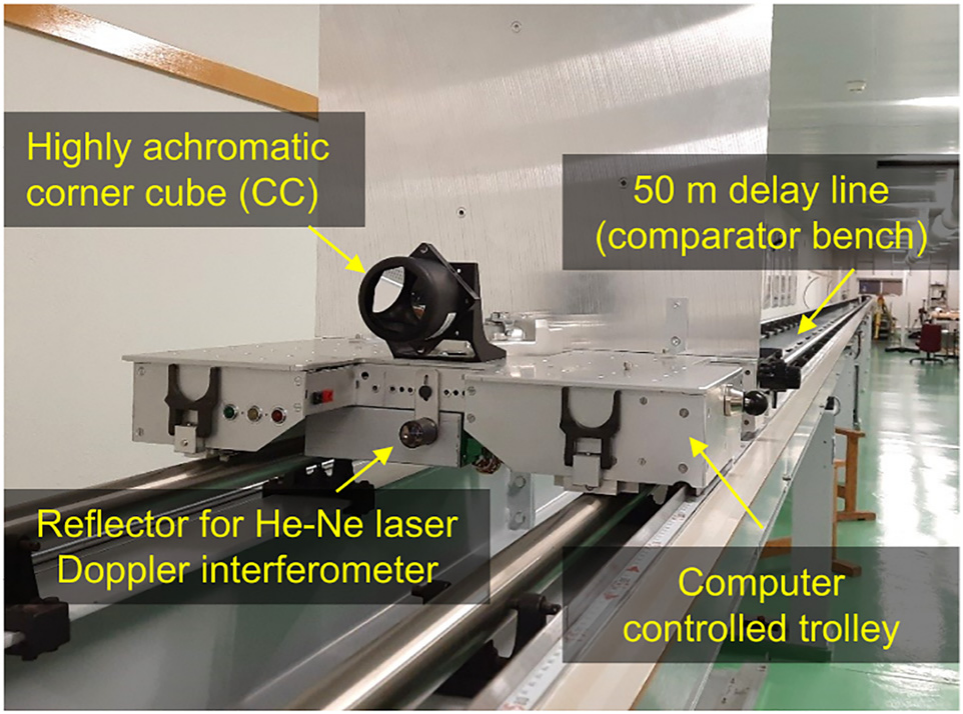
Picture of the computer-controlled trolley on a linear 50 m long comparator bench, along with the reflectors for SC-based EDM and He–Ne laser Doppler interferometer.

The measurement path is represented by a delay line formed using a moveable, highly achromatic corner cube (CC). The CC is mounted on a motorized, computer-controlled trolley that moves along a linear comparator bench. An additional reflector for a He–Ne laser Doppler interferometer (Agilent 5529A) is used as a reference for ground truth distances along the comparator, as shown in Figure 5. The experiments were conducted under stable lab conditions of 20 °C, 960 hPa, and 50% relative humidity.

## 4 Results

**Figure 6:**
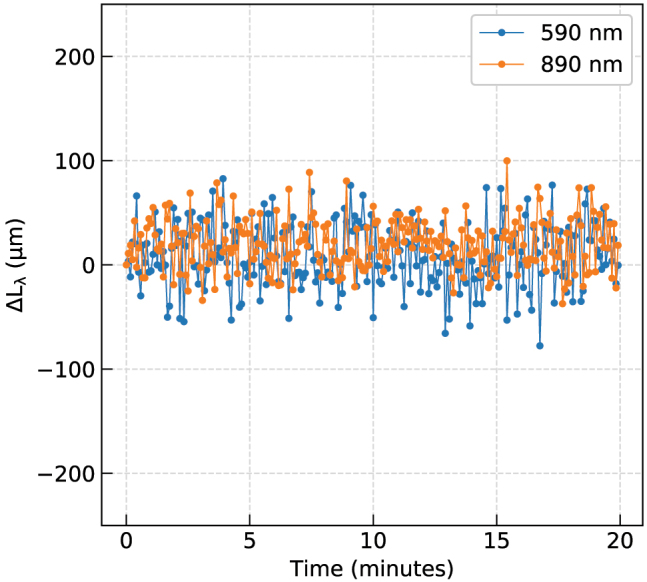
Distance deviation of individual observations averaged over 3 ms on a static reflector at 50 m.

We assessed the distance measurement precision of the above dual-wavelength EDM experimentally using measurements to the CC located approximately 50 m away from the optical bench. Distance deviations from the initial value were computed from the beat note phase observations for the 590/890 nm wavelength pair. Figure 6 shows the distance deviation (Δ*L*_*λ*_) recorded over 20 min on the two spectral bands, where each measurement is averaged over 3 ms. The measurement precision estimated from the experimental standard deviation of the distance residuals is around 30 μm for 590 nm and 25 μm for 890 nm. We chose a relatively low data averaging time of 3 ms as a practical trade-off between precision and experiment duration considering current limitations in our processing speed (extracting the I/Q phase information from the acquired samples) for real-time use.

**Figure 7:**
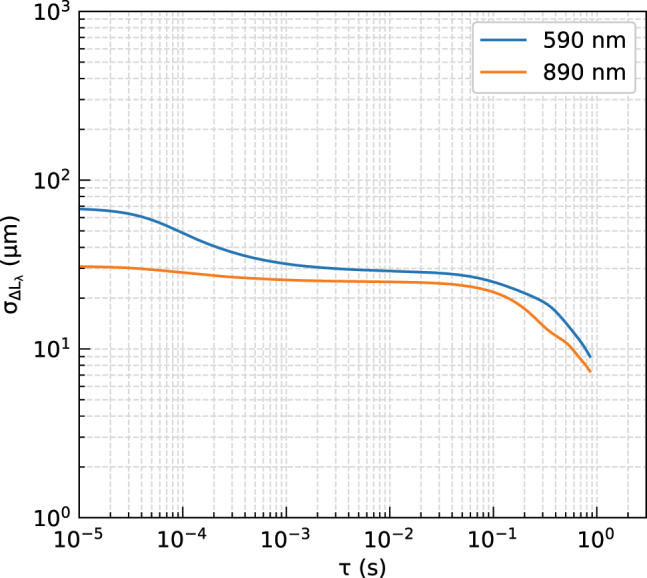
Distance deviation as a function of averaging time on a static reflector at 50 m.

We have nevertheless investigated the expected dis tance uncertainty *σ*(Δ*L*_*λ*_) for longer averaging time *τ*(*s*) on a single acquisition of 3.52 s at 1.25 GS/s. The results are obtained by computing the standard deviation of the distance time series Δ*L*_*λ*_ processed with moving average window length *τ*(*s*). The results, shown in Figure 7, indicate a slightly higher overall measurement uncertainty on the 590 nm than on the 890 nm channel, likely introduced by higher phase noise in that region on the source spectrum.

The relatively flat response below *τ* = 10^−4^ s is caused by the noise bandwidth being limited by the internal 10 kHz filter used in the digital I/Q phasemeter. The flat region between 10^−3^ s to 10^−1^ s averaging time, on the other hand, suggests the presence of flicker frequency-modulation (FM) noise, which is typically related to the physical resonance mechanism of the active oscillator or FM noise in the electronics [26]. This flat region shows no significant improvement in the measurement uncertainty with increasing averaging time from 10^−3^ s to 10^−1^ s. Beyond *τ* = 10^−1^ s, a slope of around −1 suggests that white noise dominates the frequency range in the Hz level, enabling efficient precision improvement for averaging times of some tenth of a second. Such a reduction in distance uncertainty indicates the feasibility of achieving *σ*(Δ*L*_*λ*_) better than 10 μm over 50 m by using averaging times of about 1 s, i.e., precision on the order of 0.2 ppm.

**Figure 8:**
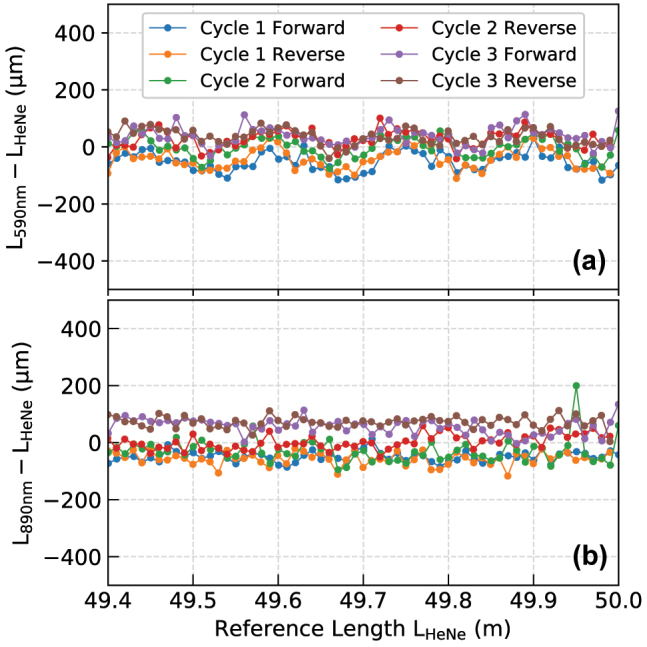
Residuals between the measured and reference (interferometer) distances for (a) 590 nm, (b) 890 nm.

Further experiments were performed to evaluate the measurement accuracy of the developed experimental platform, where the measurements obtained using our setup are compared with those from the reference interferometer. The experiments were repeated in 3 cycles at different instances of time (over 6 h), where each cycle comprises of a forward (from 50 m to 49.4 m) and reverse motion (from 49.4 m to 50 m). The range of the measurements (0.6 m) was selected to cover more than one ambiguity range of the 1 GHz beat note frequency. Defined by half of the beat note wavelength, the ambiguity range in our experiments is 15 cm, which also determines the period of the main cyclic error component. The obtained results are shown in Figure 8, where each data point represents an individual measurement averaged over 3 ms.

No further averaging could be introduced due to practical limitations related to the processing time of our setup. The obtained results are thus fully affected by the noise, and the precision in the current experiment is not better than the approximately 30 μm per channel described above. The results agree with the reference interferometer within 100 μm for both channels. Besides noise, a systematic cyclic error period of 15 cm is observed, as expected, in the 590 nm results.

Usually, such cyclic errors occur when the optical beam in the measurement path interferes with another phase-delayed component of itself (e.g., due to the presence of some spurious reflections from any optical component) or from electrical cross-talk of the photodetected beat notes before digitization. Generally, such cyclic errors are repeatable under similar measurement conditions and can be compensated through instrument calibration. On the other hand, the results obtained on 890 nm show no visible cyclic systematics, likely due to better optical isolation of this channel provided by a more efficient anti-reflection coating on the optical components.

**Figure 9:**
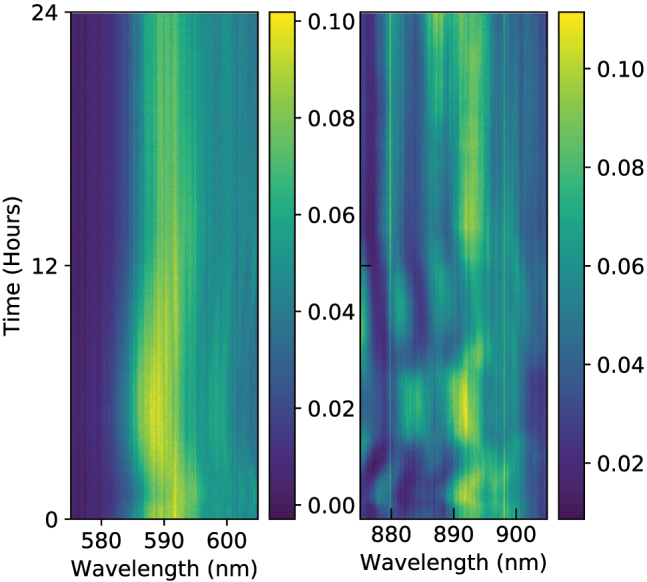
Long-term variation of the filtered supercontinuum spectrum.

Both channels show additionally slightly different bias per cycle. This shift in bias is caused by slow time-dependent drifts identified in long-term stability tests and can also be seen in these results upon careful inspection. Note the increasing bias with cycle number, which shows its correlation with time, evident in subsequent cycles 2-reverse and 3-forward of the 890 nm channel. Considering the timescale of this change (hours), we suspect this is due to the slow variations in the filtered SC power that couples into phase variations through optical power to phase coupling at the photodetector. Such effects due to power to phase coupling and pointing fluctuations have been previously reported in the literature [27] and are generally mitigated by adding an additional internal path measurement, also referred to as self-reference measurements [17, 20]. Figure 9 shows the power fluctuations of our SC laser around the selected spectrum of interest and indicates the need for such self-referenced measurements in our setup. Further work is underway to study the long-term stability by investigating the performance of a self-referenced path in our setup.

## 5 Conclusions and outlook

We have developed a supercontinuum-based EDM platform using the intermode beating approach for dual-wavelength distance measurements over 50 m. The broad spectral bandwidth of the SC offers spectral flexibility in the wavelength selection and can also be extended to multi/hyperspectral EDM.

The measurement precision is approximately 30 μm on both the 590 nm and 890 nm spectral channels over a 50 m delay line for an integration time of around 3 ms, which can be further improved to <10 μm by increasing the averaging time up to around 1 s. This represents a transfer of performance from our previously reported results [20] to a range 3 orders of magnitude longer without any degradation. Considering these performance parameters and the dispersion coefficient (|*A*| = 35) for the 590/890 nm pair, the expected measurement precision of the refractivity-compensated distance from the dual-wavelength solution would be around 35 × 10 μm = 350 μm, with promising perspectives to similar performance on longer ranges over several hundred meters.

Besides the precision results, deviations between our results using intermode beating and the ground truth distances obtained from the interferometer were less than 100 μm on relative displacements at 50 m. These results are partly affected by systematic cyclic errors that can be largely reduced by calibration and by a slow drift caused by power and pointing to phase coupling, which we expect to mitigate significantly using internal self-reference double differences.

Further work is underway to implement these mitigation strategies and investigate the long-term stability of our setup. In addition, actual inline refractivity compensated EDM will be tested along with an investigation extension to more than two wavelengths for better atmospheric compensation.
